# The Association of Rpb4 with RNA Polymerase II Depends on CTD Ser5P Phosphatase Rtr1 and Influences mRNA Decay in *Saccharomyces cerevisiae*

**DOI:** 10.3390/ijms23042002

**Published:** 2022-02-11

**Authors:** Ana I. Garrido-Godino, Abel Cuevas-Bermúdez, Francisco Gutiérrez-Santiago, Maria del Carmen Mota-Trujillo, Francisco Navarro

**Affiliations:** 1 Departamento de Biología Experimental-Genética, Universidad de Jaén, Paraje de las Lagunillas, s/n, E-23071 Jaén, Spain; aggodino@ujaen.es (A.I.G.-G.); acuevas@ujaen.es (A.C.-B.); fgutierr@ujaen.es (F.G.-S.); mcmt0005@red.ujaen.es (M.d.C.M.-T.); 2 Centro de Estudios Avanzados en Aceite de Oliva y Olivar, Universidad de Jaén, Paraje de las Lagunillas, s/n, E-23071 Jaén, Spain

**Keywords:** transcription, RNA polymerases, biogenesis, mRNA stability, Rtr1 CTD phosphatase, *Saccharomyces cerevisiae*

## Abstract

Rtr1 is an RNA polymerase II (RNA pol II) CTD-phosphatase that influences gene expression during the transition from transcription initiation to elongation and during transcription termination. Rtr1 interacts with the RNA pol II and this interaction depends on the phosphorylation state of the CTD of Rpb1, which may influence dissociation of the heterodimer Rpb4/7 during transcription. In addition, Rtr1 was proposed as an RNA pol II import factor in RNA pol II biogenesis and participates in mRNA decay by autoregulating the turnover of its own mRNA. Our work shows that Rtr1 acts in RNA pol II assembly by mediating the Rpb4/7 association with the rest of the enzyme. *RTR1* deletion alters RNA pol II assembly and increases the amount of RNA pol II associated with the chromatin that lacks Rpb4, decreasing Rpb4-mRNA imprinting and, consequently, increasing mRNA stability. Thus, Rtr1 interplays RNA pol II biogenesis and mRNA decay regulation. Our data also indicate that Rtr1 mediates mRNA decay regulation more broadly than previously proposed by cooperating with Rpb4. Interestingly, our data include new layers in the mechanisms of gene regulation and in the crosstalk between mRNA synthesis and decay by demonstrating how the association of Rpb4/7 to the RNA pol II influences mRNA decay.

## 1. Introduction

RNA polymerase II (RNA pol II) is composed of a 10-subunit core polymerase and the dissociable heterodimer Rpb4 and Rpb7 in *Saccharomyces cerevisiae* [1,2,3]. Rpb4/7 participates in different steps of transcription, such as initiation, elongation, termination, and polyadenylation, as well as gene looping and dephosphorylation of the CTD of Rpb1 [4,5,6,7,8,9,10]. Furthermore, Rpb4/7 binds mRNA cotranscriptionally, influencing mRNA export, translation, and decay [11,12,13,14,15,16,17,18]. Rpb4 and Rpb7 act as RNA binding proteins (RBP) and associate with regulatory factors such as Pat1, Lsm2, Puf3 or Not5 to link mRNA synthesis and decay [14,15,16,19,20]. Interestingly, post-translational modifications of Rpb4/7 are required to connect transcription with post-transcriptional mechanisms [21,22].

It is assumed that gene expression ensures appropriate mRNA levels, which maintains mRNA homeostasis by coordinating transcription in the nucleus, and mRNA decay, which occurs mainly in the cytoplasm [23,24,25,26]. Some authors define gene expression as a circular process during which the synthesis and degradation of mRNAs are coordinated in a process called the crosstalk of mRNA, which extends the classic view of the Central Dogma of the Molecular Biology [13,18,25,27,28,29,30]. During this process, the mRNA decay machinery may influence transcription and the transcription machinery might regulate mRNA fate [18,25,28,30,31]. However, despite the importance of the dimer Rpb4/7 in both processes, the impact that the Rpb4/7 assembly/association with the rest of the enzyme has on mRNA decay (and thus, also on its stability) was barely explored.

Assuming that biogenesis of eukaryotic RNA pol II is similar to the bacterial one [32], three subassembly complexes must be formed: the Rpb1 (composed of Rpb1, Rpb5, Rpb6 and Rpb8), the Rpb2 (consisting of Rpb2 and Rpb9), and the Rpb3 (comprising Rpb3, Rpb10, Rpb11, and Rpb12). These subcomplexes interact to form a 10-subunit core that finally incorporates the heterodimer Rpb4/7 to form the complete enzyme [32]. Some factors participate in the assembly of RNA pol II in yeast, including Rtp1, Rba50 and the small GTPases Gpn1 (Npa3), Gpn2, and Gpn3 [33,34,35,36,37], as well as the small GTPases orthologues, the HSP90 cochaperone, and the R2TP/prefoldin-like complex in human cells [38,39]. Notably, Npa3 purification leads to the identification of the RNA pol II lacking Rpb4/7 [40]. Most of these factors are specific for RNA pol II assembly, although small GTPases Gpn2 and Gpn3 also mediate the assembly of RNA pol III [35,41]. Prefoldin-like Bud27 participates in the assembly of the three RNA pols by mediating the association of Rpb5 and Rpb6 with the enzymes prior to their nuclear import [42,43]. In addition to RNA pol II assembly factors, in *S. cerevisiae* other proteins participate in RNA pol II transport to the nucleus, such as Iwr1 [44,45], Rtp1 [34], and Rtr1 [45]. The nuclear import of RNA pol II in *S. cerevisiae* occurs mainly through the Iwr1-dependent process [44,45]. However, the largest RNA pol II subunits can also be imported to the nucleus by an Iwr1-independent pathway with the participation of Rtr1 and Rtp1 and the action of microtubules [45]. This Iwr1-independent pathway also seems to operate for the smallest RNA pol II subunits, which can enter the nucleus by diffusion [45].

Rtr1 (for “regulator of transcription” 1) and its human orthologue RPAP2 (for “RNAPII-associated polypeptide”) were initially described as RNA polymerase II interactors [46,47]. Rtr1 was initially defined as an S5-P CTD phosphatase with a proposed role in the transition from transcription initiation to elongation in vivo [48,49]. Furthermore, Rtr1 can dephosphorylate Tyr1-P CTD in vitro, which suggests a role in the transcription elongation and termination steps [50]. In addition, Rtr1 was proposed to associate with RNA pol II during transcription depending on the CTD phosphorylation state, and this interaction would have an influence on Rpb4/7 dissociation from the rest of the enzyme [40].

Despite the effect of Rtr1 in transcription, some studies proposed a role for Rtr1 and RPAP2 in the biogenesis of RNA pol II by acting as import factors to allow RNA pol II shuttling from the cytoplasm to the nucleus [45,51]. Rtr1 shuttles between the nucleus and cytoplasm [46], with a major cytoplasmic localization [46,52,53], and similarly, human RPAP2 can be found in both the nucleus and cytoplasm [51]. Interactions between Rtr1 or RPAP2 with RNA pol II are controversial. Some authors propose the interaction between Rtr1 and active phosphorylated RNA pol II [46,48,54,55], while others posit that the efficient RNA pol II/RPAP2 interaction does not require the CTD domain of the enzyme in either in vitro or in vivo [51]. RPAP2 interacts with RNA pol II in vitro [51], associates with the Rpb2 and Rpb3 subassembly complexes [38,56], and directly binds the Rpb5 and Rpb6 subunits of the enzyme [57,58]. Furthermore, RNA pol II purification using Rtr1-TAP as bait results in the isolation of a 10-subunit enzyme with a relative depletion of the heterodimer Rpb4/7 [40]. In line with the role of Rtr1 as an RNA pol II transporting factor, the interaction between Rtr1 and the nucleocytoplasmic transport protein Ran was described [59]. Furthermore, in Rtr1 purification experiments, interactions with GTPases Gpn3 and Npa3 were detected [40,54]. Note that Npa3 was implicated in RNA pol II nuclear import regulation [36]. In addition, Rtr1 might return to the cytoplasm in association with Npa3 [51,60], and the human RPAP2 with the Npa3 orthologue GPN1 [47,51].

It was proposed that Rtr1 plays a role in mRNA stability by autoregulating the stability of its own mRNA, as a result of their mutual interaction, on a degradation pathway that involves 5′-3′ DExD/H-box RNA helicase Dhh1 and 3′-5′ exonucleases Rex2p and Rex3p [61].

This work suggests that Rtr1 participates in mRNA crosstalk and may influence the mRNA life cycle from transcription to mRNA decay. However, the specific role of Rtr1 in both assembly and mRNA decay regulation is poorly understood. In this work we provide evidence that Rtr1 acts in the assembly/association of the dimer Rpb4/7 with the RNA pol II in the cytoplasm, althougt we cannot discard additonal association in the nucleus.

Our results show that the absence of Rtr1 causes RNA pol II assembly defects, leading to a decrease in the amount of chromatin-associated RNA pol II containing the Rpb4 subunit. Thus, the absence or Rtr1 affects Rpb4-mRNA imprinting and increases mRNA stability, in agreement with alterations of RNA pol II assembly causing the dissociation of Rpb4 from the rest of the complex [13,17]. We discard a major role of Rtr1 in Rpb4 dissociation from the rest of the enzyme during the transcriptional process. Our data also suggest that Rtr1 plays a specific role in mRNA decay regulation, and that there is a coordinated cooperation between Rtr1 and Rpb4 to modulate mRNA imprinting and mRNA decay. 

## 2. Results

### 2.1. The Presence of Rpb4 in the Chromatin-Associated RNA Polymerase II Depends on Rtr1

Our previous work demonstrated that *RTR1* deletion is lethal when combined with either of the *RPB1* foot mutations, *rpo21-4* or *rpb1-84*, both of which provoke the disassembly of dimer Rpb4/7 from the rest of the enzyme [62]. Interestingly, we identified *RTR1* as a multicopy suppressor of the temperature sensitivity phenotype of the mutant *rpo21-4* (not shown). In addition, as indicated by quantitative proteomic analyzes, Rpb4 and Rpb7 would dissociate from the rest of RNA pol II during transcription elongation [40], most likely with the participation of Rtr1, whose association with the enzyme could require Ser2 CTD phosphorylation [54]. Furthermore, Rtr1 and its human counterpart RPAP2 participate in the biogenesis of RNA pol II by facilitating its transport from the cytoplasm to the nucleus [45,51].

As such, we wondered if Rtr1 would be required for the assembly or the association of the Rpb4/7 dimer to the rest of the RNA pol II bound to chromatin.

**Figure 1:**
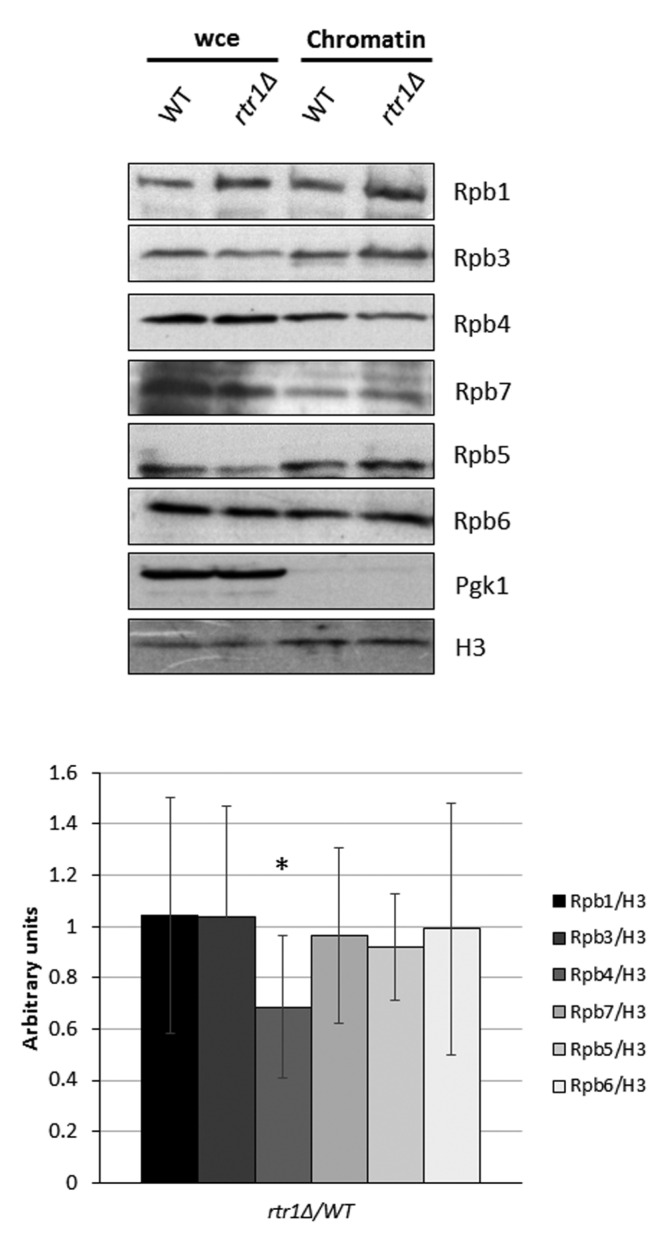
Analysis of chromatin-associated RNA pol II. Whole-cell extract and chromatin-associated proteins isolated by yChEFs procedure [63,64] from wild-type and *rtr1Δ* strains grown in YPD medium at 30 °C were analyzed by Western blot with specific antibodies against Rpb1 (y-80), Rpb3, Rpb4, Rpb7, Rpb5, and Rpb6. H3 histone was used as a positive control of chromatin-associated proteins, and Pgk1 was negative control of cytoplasmic contamination. Lower panel: quantification from chromatin fractions in upper panel, for each RNA pol II subunit vs. histone H3, between *rtr1Δ* mutant and wild-type strains. Data are median and standard deviation of at least three independent biological replicates. * *p* < 0.05 (*t*-test).

To explore whether Rtr1 could impact the presence of Rpb4/7 in the chromatin-associated RNA pol II complexes, likely engaged in transcription, we analyzed the RNA pol II associated with chromatin. For this purpose, we isolated chromatin-enriched fractions with their associated proteins, using the yChEFs procedure [63,64], in both *rtr1Δ* cells and the wild-type isogenic strain (Figure 1 and Figure S1). We analyzed, by Western blot with specific antibodies, the amount of Rpb4 and Rpb7 in the chromatin relative to Histone H3 (Figure 1), and Rpb1 (Figure S1) with an anti-Rpb1 antibody against the amino-terminal domain of the largest subunit of the RNA pol II (amino acids 1 to 80) to avoid interference with Rpb1 phosphorylation. The absence of the cytoplasmic 3-phosphoglycerate kinase (Pgk1) in the enriched chromatin-fractions, and the similar levels of histone H3 in samples from wild-type and mutant cells, indicated the successful isolation of and similar chromatin levels in both samples. As observed, the amount of Rpb4 associated with chromatin significantly diminished in the *rtr1Δ* strain in relation to the wild-type strain (Figure 1). However, the levels of Rpb7 associated with the chromatin were similar in both the wild-type and *rtr1Δ* mutant cells, as is the case for other subunits of the RNA pol II, Rpb1, the α-subunit Rpb3, and the Rpb5 and Rpb6 subunits. Accordingly, the Rpb4/Rpb1 ratio decreased in the absence of Rtr1 (Figure S1). These results suggest that in the absence of Rtr1, the levels of RNA pol II lacking Rpb4 in chromatin increase and coexist with complete RNA pol II.

**Figure 2:**
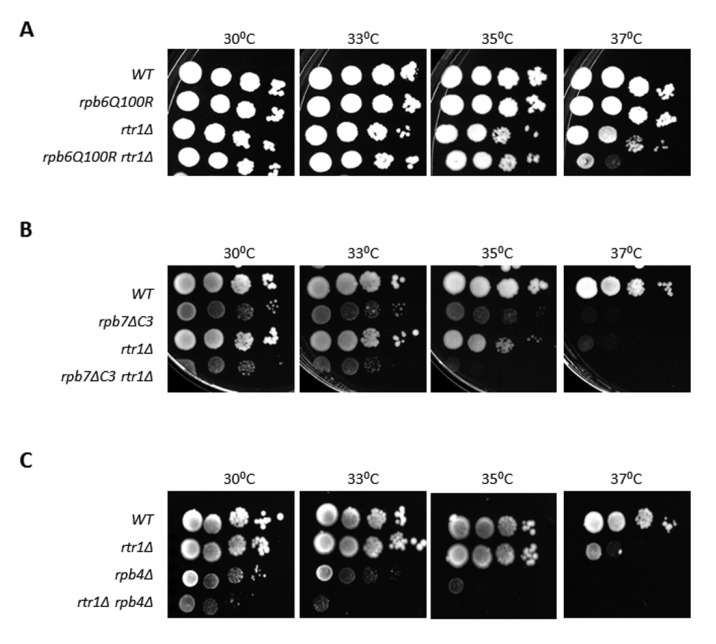
Genetic interactions between *RTR1* and *RPB6*, *RPB7,* and *RPB4*. (**A**) *rtr1Δ* and *rpb6Q100R* single and double mutants grown at different temperatures in YPD medium. (**B**,**C**) growth of *rtr1Δ* and *rpb7ΔC3* (**B**) or *rtr1Δ* and *rpb4Δ* (**C**) single and double mutants in YPD medium at different temperatures.

A functional relationship between Rtr1 and the correct association of Rpb4/7 with the RNA pol II is proposed based on genetic interactions between mutant alleles *rtr1Δ* and *rpo21-4* or *rpb1-84* (*RPB1* gene), which cause the dimer Rpb4/7 to dissociate from the rest of the enzyme [62]. We further investigated this hypothesis by analyzing the genetic interactions between *RTR1* and *RPB6* by combining the *rtr1Δ* mutation with the *rpb6Q100R* mutation causing the loss of dimer Rpb4/7 from the rest of RNA pol II [65], (Figure 2A). This study was extended to the *RPB7* mutation *rpb7ΔC3,* which impairs the association of Rpb4 with the RNA pol II [8], (Figure 2B), and to the *rpb4Δ* mutation as a control [46], (Figure 2C). As expected, strong genetic interactions were observed in all cases under stress conditions (high temperature).

Based on these data, we propose a role for Rtr1 in the Rpb4 association with RNA pol II. However, we cannot discard the possibility that it might have an impact on Rpb4 dissociation during transcription in the *rtr1Δ* mutant cells, in line with previous data [40].

### 2.2. Lack of Rtr1 Does Not Account for a Global Effect on Rpb4 Dissociation from RNA Pol II during Transcription Elongation

It was proposed that Rpb4 and Rpb7 dissociate from the rest of RNA pol II during transcription elongation, likely with the participation of Rtr1 [54].

**Figure 3:**
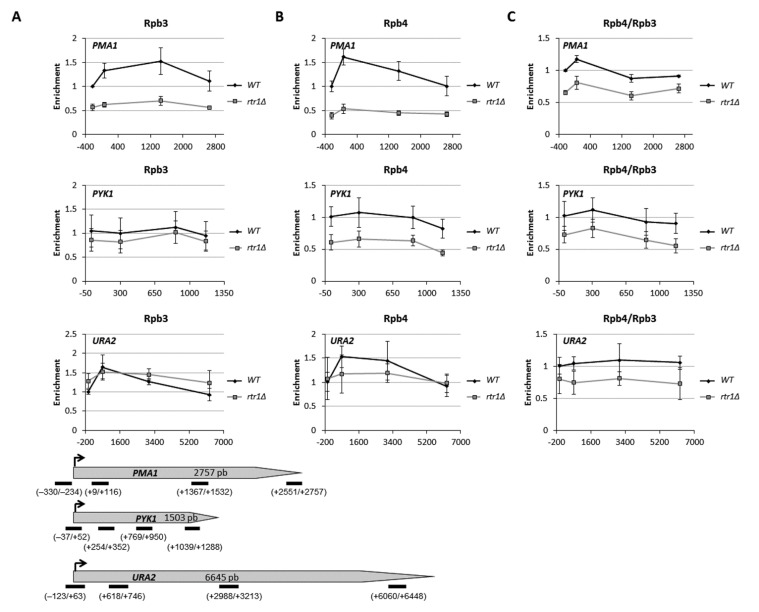
*rtr1Δ* mutation affects gene occupancy by RNA pol II, but not global Rpb4 dissociation. Chromatin immunoprecipitation (ChIP) analysis for different genes in wild-type and *rtr1Δ* cells, performed with anti-Rpb3 (**A**) and anti-Rpb4 (**B**) antibodies, against Rpb3 and Rpb4 RNA pol II subunits. (**C**): Rpb4/Rpb3 ratios for Rpb4 dissociation analysis, from left and middle panel’s results. Lower panel: transcription units used in this work indicating location of analyzed PCR amplicons. Values found for immunoprecipitated PCR products were compared to those of total input, and ratio of each PCR product of transcribed genes to a nontranscribed region of chromosome V was calculated.

To investigate whether the decrease in Rpb4 associated to the chromatin when Rtr1 was absent could be the consequence of an increase in Rpb4 dissociation during transcription, we explored, by ChIP, the Rpb3 and Rpb4 occupancy along the entire length of different transcriptional units in the wild-type and *rtr1Δ* mutant cells. As shown in Figure 3, Rpb3 occupancy increased from the promoter region to halfway or the beginning of *PMA1* and *PYK1* ORFs, respectively, and decreased through the 3′ regions in the wild-type and *rtr1Δ* mutant cells. In addition, the lack of Rtr1 diminished Rpb3 occupancy for the entire transcriptional units in relation to the wild-type cells. These results fall in line with those previously reported [48]. Notably, Rpb4 occupancy showed a similar profile to Rpb3 occupancy in the wild-type and *rtr1Δ* mutant cells, peaking at the beginning of genes *PMA1* and *PYK1,* and decreasing through the 3′ regions (Figure 3). However, Rpb4 occupancy drastically decreased for each analyzed region in relation to the wild-type cells when Rtr1 was lacking (Figure 3). The dissociation of Rpb4 from the rest of RNA pol II during transcription was represented by the ratio between Rpb4 and Rpb3 occupancies along the entire length of the transcriptional units (Figure 3). As shown, the Rpb4/Rpb3 ratios for *PMA1* and *PYK1* lowered from the beginning to the 3′ region of these genes in the wild-type cells, which supports Rpb4 dissociation occurring [6,10,40]. Strikingly, no major differences in the Rpb4/Rpb3 profile along the *PMA1* and *PYK1* transcription units were observed in the *rtr1Δ* mutant cells in relation to the wild-type strain, which suggests that the lack of Rtr1 did not significantly impact the global Rpb4 dissociation. However, as expected from data above, the Rpb4/Rpb3 ratios lowered for each analyzed region along the transcriptional units in the *rtr1Δ* mutant cells, which indicates that the drop of Rpb4 vs. the rest of RNA pol II observed in chromatin fractions (Figure 1 and Figure S1) was primarily a consequence of a defect in Rpb4 association with the rest of the enzyme, and not of Rpb4 dissociation during transcription. Similar results, albeit with differences in the Rpb3 and Rpb4 occupancy profiles between the wild-type and *rtr1Δ* mutant cells, were also observed for the *URA2* transcription unit (Figure 3). Finally, we did not observe a decrease in the Rpb4/Rpb3 ratio for the *MTG1* gene (Figure S2), which could fall in line with data that propose the influence of Rtr1 and other elongation factors on Rpb4 dissociation during transcription only for a limited number of genes [40].

Taken together, our data indicate that the decrease in Rpb4 bound to the chromatin observed in the absence of Rtr1 is not mainly caused by Rpb4 dissociation from the RNA pol II during transcription elongation, and instead point to Rtr1 mediating RNA pol II biogenesis.

### 2.3. Rtr1 Mediates the Correct Assembly of the RNA Polymerase II

**Figure 4:**
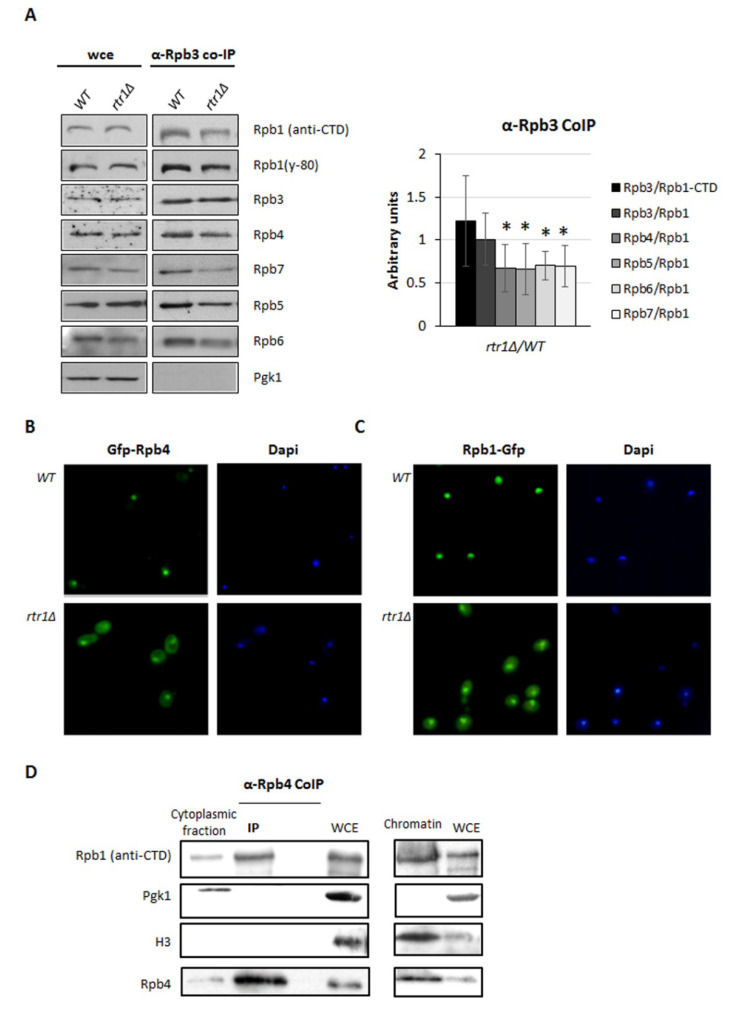
*RTR1* deletion affects assembly of RNA pol II. (**A**) RNA pol II immunoprecipitated from wild-type and *rtr1Δ* mutant strains using an anti-Rpb3 antibody and grown in YPD medium at 30 °C. Upper panel shows Western blot analysis of subunits Rpb1 (anti-CTD and y-80 antibodies), Rpb3, Rpb4, Rpb7,Rpb5, and Rpb6 of RNA pol II in whole-cell extracts, and in immunoprecipitated samples. Right panel: quantification of Western blot showing *rtr1Δ* mutant/wild-type strains ratio for each subunit vs. Rpb1 (y-80). Pgk1 was tested as a negative control in RNA pol II purified samples. Graphs represent median and standard deviation of at least three independent biological replicates.* *p* < 0.05 (*t*-test). (**B**) Live-cell imaging of Gfp-Rpb4 in wild-type and *rtr1Δ* mutant strains. (**C**) Live-cell of Rpb1-Gfp localization in wild-type and *rtr1Δ* mutant strains. (**D**) RNA pol II immunoprecipitated from cytoplasmic-enriched fractions and RNA pol II in chromatin-enriched fractions obtained by yChEFs procedure [63], with an anti-Rpb4 antibody from a wild-type strain grown in YPD medium at 30 °C. Western blot analysis of Rpb1 (anti-CTD antibody) and Rpb4 subunits of RNA pol II. H3 histone was used as a positive control for chromatin-associated proteins, and Pgk1 for cytoplasm.

In an attempt to decipher whether Rtr1 mediates the assembly of Rpb4 with the rest of RNA pol II, we performed protein immunoprecipitation with an anti-Rpb3 antibody against the Rpb3 subunit of RNA pol II in an *rtr1Δ* mutant and its isogenic wild-type strain. These experiments allowed us to analyze the total RNA pol II and not only the chromatin-associated enzyme. Then, we analyzed the composition of the enzyme by Western blot, with antibodies against several RNA pol II subunits. The results showed a significant decrease in the Rpb4/Rpb1 ratio in the above-indicated immunoprecipitation assays (Figure 4A and Figure S3) and also in Rpb7, Rpb5, and Rpb6 vs. Rpb1 (Figure 4A and Figure S3). In contrast, the lack of Rtr1 did not show a significant change in the association between Rpb3 and Rpb1. Similar results were obtained when a polyclonal antibody against the C-terminal domain (CTD) of Rpb1 [63] was used (Figure 4A and Figure S3).

The deletion of *RTR1* in yeast or human RPAP2 silencing cause the partial cytoplasmic mislocalization of Rpb1 [45,51]. Accordingly, we analyzed if *RTR1* deletion would also provoke the cytoplasmic accumulation of Rpb4. To do so, we analyzed by fluorescent microscopy, the Rpb4 localization in *rtr1Δ* mutant and wild-type strains that express GFP-Rpb4. As shown in Figure 4B, Rpb4 clearly accumulated in both the cytoplasm and nucleus of *rtr1Δ* mutant cells, while only nuclear localization was observed in the wild-type cells. As expected, Rpb1-GFP mislocalization was observed in the *rtr1Δ* mutant with respect to the wild-type, corroborating previously described results [45] (Figure 4C).

Taken together, our data suggest a role for Rtr1 in RNA pol II assembly. Moreover, as only Rpb4 decreased in the chromatin-associated RNA pol II, our results point to the participation of Rtr1 in the assembly of Rpb4/7, leading to the nuclear shuttling of two active RNA pol II populations, one complete and the other lacking Rpb4, given that the RNA pol II lacking Rpb7 or Rpb4/7 is not functional [66]. Furthermore, according to the localization of Rpb4, these data suggest that the assembly of Rpb4/7 to the RNA pol II may most likely occur in the cytoplasm.

We obtained cytoplasm-enriched fractions from wild-type cells using yChEFs methodology [63] to better define whether Rpb4/7 assembly occurs in the cytoplasm. Using these fractions, we affinity purified Rpb4 with anti-Rpb4 antibodies and analyzed the purified samples using Western blot with antibodies against the Rpb1 subunit of RNA pol II. We observed the enrichment of Rpb1 in the Rpb4 purified samples (Figure 4D). As a control, chromatin-enriched fractions corroborated previous results showing the presence of RNA pol II in the chromatin (Figure 1). Similar results were obtained by performing Rpb3 immunoprecipitation (results not shown). Taken together, these results suggest that Rpb4, and then Rpb4/7, assembles with the RNA pol II in the cytoplasm, although we cannot discard additional association in the nucleus.

### 2.4. Defective Association of Rpb4 with the RNA Pol II in the rtr1Δ Mutant Affects mRNA Stability

It was demonstrated that Rpb4 imprints mRNA in the context of transcribing RNA pol II and regulates mRNA decay in an Rpb7-dependent manner. Consequently, the decrease in Rpb4-mRNA imprinting increases mRNA stability [11,13,15,17,26,67,68].

**Figure 5:**
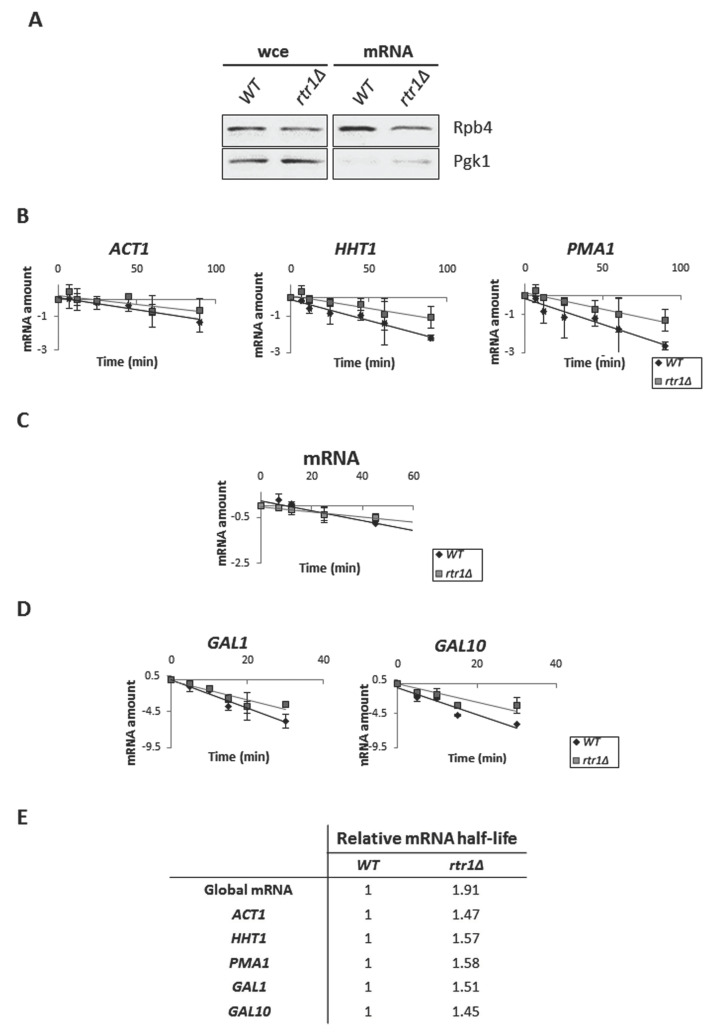
*rtr1Δ* decreases Rpb4-mRNA imprinting and increases mRNA stability. (**A**) Western blot of Rpb4 in both whole-cell free extracts and oligo-dT purified mRNAs after exposure to 1200 mJ/cm^2^ of 254 nm UV in wild-type and *rtr1Δ* mutant. Anti-Pgk1 antibody was used as a negative control. (**B**) mRNA levels, measured by RT-qPCR, for genes *ACT1, HHT1,* and *PMA1* after thiolutin addition to block transcription in wild-type and *rtr1Δ* mutant. (**C**) Global mRNA levels measured at different times after thiolutin addition to block transcription in wild-type and *rtr1Δ* mutant using an oligodT probe by a dot-blot assay. (**D**) *GAL1* and *GAL10* mRNA amounts measured by RT-qPCR, in wild-type and *rtr1Δ* mutant at different times after blocking transcription with glucose. Time 0 corresponded to cells grown in presence of galactose as carbon source. In (**B**–**D**), drop in mRNA levels after shutoff at different times is represented on a natural logarithmic scale. In (**B**,**D**), rRNA 18S was used as a normalizer. (**E**) Relative mRNA half-lives calculated from experiments represented in (**B**–**D**). All experiments corresponded to at least three independent biological replicates.

Accordingly, we hypothesized that the defect in the assembly/association of Rpb4 with the RNA pol II caused by the absence of Rtr1 could impact Rpb4-mRNA imprinting and, consequently, mRNA stability. To investigate this possibility, we analyzed whether the absence of Rtr1 could alter the association of Rpb4 with mRNAs. To do so, we crosslinked the proteins bound to mRNA by using UV irradiation at 254 nm, in *rtr1Δ* mutant and wild-type cells, as previously described [13]. Then, we analyzed the association of Rpb4 with mRNAs in total poly(A)-containing mRNA, using Western blot with specific antibodies. As shown in Figure 5A, *RTR1* deletion reduced the amount of Rpb4 associated with mRNA in relation to its wild-type isogenic strain. This result indicates that Rtr1 influences Rpb4-mRNA imprinting, a process that may depend on the amount of Rpb4 bound to the chromatin-associated RNA pol II, and that may occur cotranscriptionally, as previously described [13,26]. In addition, no major cytoplasmic contamination was observed, as indicated the low amount of Pgk1 protein in the samples (Figure 5A).

To explore if the reduction in Rpb4-mRNA imprinting affected mRNA stability, we treated the cultures of the wild-type and *rtr1Δ* cells with 5 µg/mL thiolutin to block transcription and total RNA was extracted at different times after thiolutin addition, as previously described [13]. The RT-qPCR analysis of the mRNA levels for genes *ACT1*, *HHT1* and *PMA1* demonstrated an increase in the mRNA half-life for all the tested genes in the *rtr1Δ* mutant in relation to the wild-type strain (Figure 5B,E). The global mRNA decay analysis, by dot-blot using a fluorescent oligo-dT probe, corroborated these results by revealing an increase in global mRNA half-life of 1.91-fold in the mutant *versus* the wild-type strain (Figure 5C,E). Finally, we analyzed *GAL1* and *GAL10* genes mRNA decay by shifting cells cultivated in minimal medium containing galactose as a carbon source to glucose to stop transcription (Figure 5D,E). Our data showed an increase in *GAL1* and *GAL10* mRNA half-life in the *rtr1Δ* mutant, corroborating data above. However, our data cannot discard a direct effect of Rtr1 in basal transcription.

**Figure 6:**
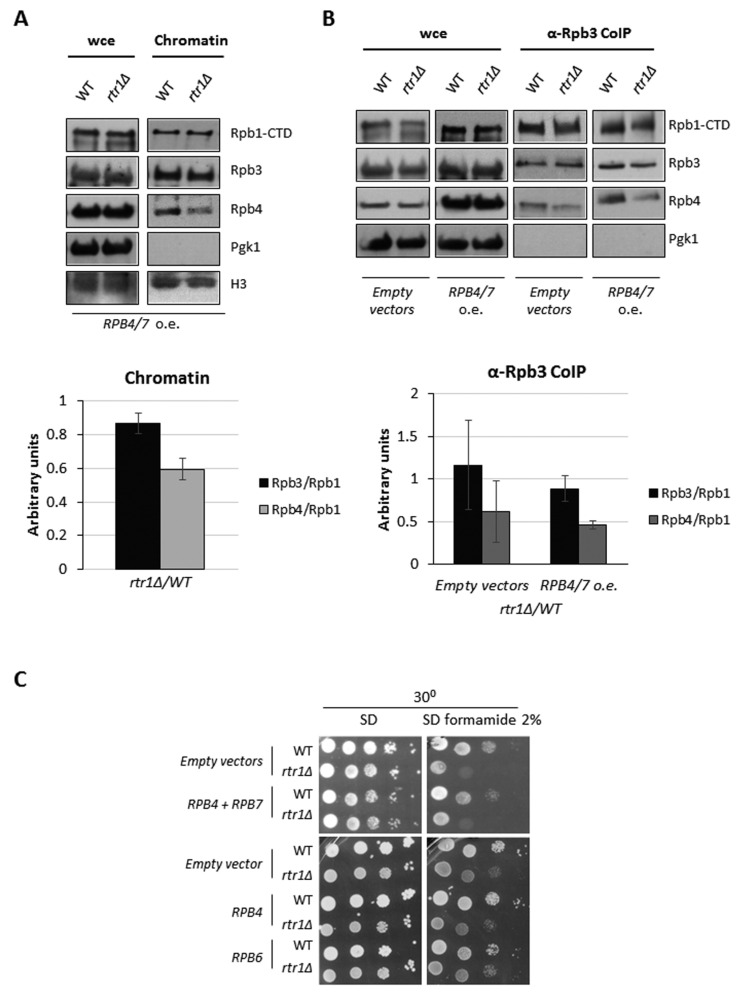
*RPB4/7* overexpression does not overcome RNA pol II assembly defect in *rtr1Δ* mutant. (**A**) whole-cell extract and chromatin enriched fractions obtained by yChEFs procedure [63,64] from wild-type and *rtr1Δ* cells overexpressing *RPB4/7* genes from high copy number plasmids, grown in SD medium at 30 °C. Proteins were analyzed by Western blot with specific antibodies against Rpb1, Rpb3, and Rpb4. H3 histone was used as a positive control of chromatin-associated protein and Pgk1 employed as a negative control of cytoplasmic contamination. Lower panel: quantification for each RNA pol II subunit vs. Rpb1. Median and standard deviation of two independent biological replicates. (**B**) Rpb3 immunoprecipitation in wild-type and *rtr1Δ* cells both overexpressing *RPB4/7* genes from high copy number plasmids or harboring corresponding empty vectors, grown in SD medium at 30 °C. Western blots of Rpb1, Rpb3, Rpb4, and Pgk1 from whole cell crude extracts and immunoprecipitated samples are shown (upper panel). Lower panel: quantification of Western blot showing *rtr1Δ* mutant/wild-type strains ratio for each subunit vs. Rpb1. Median and standard deviation of two independent biological replicates. (**C**) *RPB4* + *RPB7*, *RPB4* and *RPB6* overexpression in wild-type and *rtr1Δ* mutant cells grown in SD and SD supplemented with 2% formamide at 30 °C.

However, *RPB4 + RPB7* overexpression overcame neither the differences in Rpb4 bound to the chromatin-associated RNA pol II observed in the *rtr1Δ* mutant cells in relation to a wild-type strain (Figure 6A), nor the global association of Rpb4 with RNA pol II (Figure 6B). These results are in line with the inability of *RPB4/7* overexpression to suppress the sensitivity of the *rtr1Δ* mutant to 2% formamide phenotype (Figure 6C), [46]. Accordingly, overexpression of *RPB4*, or *RPB6*, the gene coding for the Rpb6 subunit that connects dimer Rpb4/7 with the core of RNA pol II [42,62,69], did not overcome the sensitivity to the 2% formamide phenotype of the *rtr1Δ* mutant (Figure 6C). These results agree with the role of Rtr1 in several steps of RNA pol II transcription [48,49,50,70].

All these data indicate that Rpb4 plays a direct role in mRNA imprinting and decay in the *rtr1Δ* mutant cells, as the alteration of the Rpb4 association with the RNA pol II would affect the amount of chromatin-associated RNA pol II containing Rpb4, and would consequently decrease Rpb4-mRNA imprinting, thus increasing mRNA stability. Indeed, similar results were previously demonstrated for other *rpb1* and *rpb6* mutants where defects in RNA pol II integrity alter the correct association of the dimer Rpb4/7 [13,17,62,71].

### 2.5. Rtr1 Cooperates with Rpb4 for mRNA Imprinting

**Figure 7:**
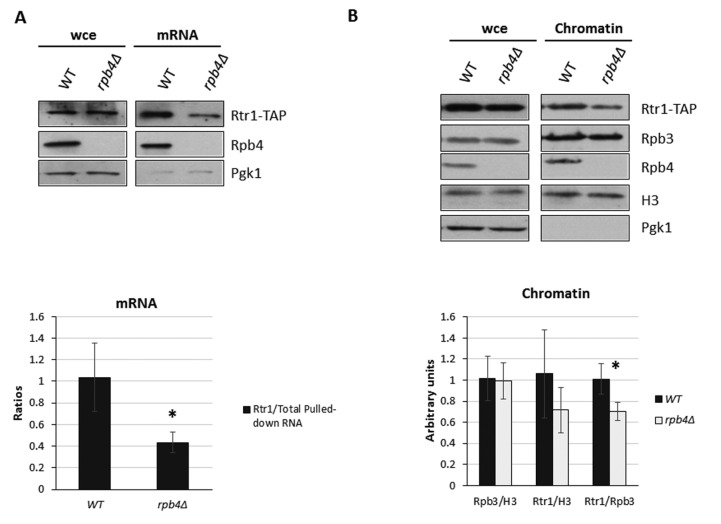
Rtr1 and Rpb4 cooperate to control mRNA stability. (**A**) Western blot of whole-cell crude extracts and oligo-dT purified mRNAs after exposure to 1200 mJ/cm^2^ of 254 nm UV in wild-type and *rpb4Δ* mutant grown in SD medium at 30 °C. Pgk1 was used as a negative control of mRNA-associated proteins. (**B**) Western blot of whole-cell extracts and chromatin-enriched fractions obtained by yChEFs procedure [63,64] from *rpb4Δ* mutant and its isogenic wild-type strain grown in YPD medium at 30 °C. A similar amount of chromatin was purified from wild-type and mutant strains as similar levels of histone H3 were detected. Pgk1 was used as a negative control of cytoplasmic contamination. Rpb4 was detected by a specific anti-Rpb4 antibody and Rtr1 was detected using an anti-TAP antibody in strains containing an *rtr1::TAP* allele. All experiments corresponded to at least three independent biological replicates. * *p* < 0.05 (*t*-test).

Our above results revealed generally increased mRNA stability in *rtr1Δ* mutant cells in correlation with diminished Rpb4-mRNA imprinting, which likely resulted from a defect in Rpb4 binding to chromatin-associated RNA pol II. Given the results describing that impairing Rpb4 association with mRNAs would lead to reduced mRNA decay [13,71] and that Rtr1 physically interacts with its own mRNA by autoregulating its turnover [61], we investigated the functional relation between Rtr1 and Rpb4, and analyzed whether Rpb4 would also affect the association between Rtr1 and mRNA. To this end, we performed UV-crosslinking and mRNA isolation in the *rpb4Δ* mutant and its wild-type isogenic strains, both containing a functional Rtr1-TAP tagged version of this protein. Surprisingly, Rtr1 globally associated with mRNAs in both the wild-type and *rpb4Δ* mutant cells (see Figure 7A). Notably, *RPB4* deletion decreased the Rtr1 association with mRNAs, which indicates that the lack of Rpb4 affects Rtr1-mRNA imprinting.

Considering that the interaction of Rpb4 with mRNA occurs in the context of RNA pol II during transcription [13,17] and that Rtr1 also associates with the transcribing RNA pol [48,50], we speculated that Rpb4 and Rtr1 could mutually impact their association with chromatin and, consequently, mRNA imprinting. According to our data above, which revealed a reduction in the Rpb4 association with chromatin as a result of *RTR1* deletion, we analyzed the association of Rtr1 with chromatin. To do so, we purified chromatin fractions using the yChEFs approach [63,64] from Rtr1-TAP tagged *rpb4Δ* mutant and its wild-type isogenic strain, and analyzed the presence of Rtr1 by Western blot (Figure 7B, top panel). We observed that the lack of Rpb4 lowered the levels of Rtr1 associated with chromatin, without any major effect on global Rtr1 in whole-cell lysates. Taken together, our data indicate that Rtr1 and Rpb4 cooperate to modulate their association with chromatin, and concomitantly, their mRNA imprinting, which likely occurs cotranscriptionally.

## 3. Discussion

This work unravels the role of RNA pol II CTD Ser5-P phosphatase Rtr1 in the association of Rpb4 with the RNA pol II and in mRNA decay and indicates that Rtr1 interconnects both processes. The effect of Rtr1 on the correct assembly of Rpb4 (and probably of the dimer Rpb4/7) with the RNA pol II, likely in the cytoplasm, would determine the amount of chromatin-associated RNA pol II population containing Rpb4, which would, in turn, cotranscriptionally impact mRNA decay. Our data also suggest that Rtr1 cooperates with Rpb4 to mediate the mRNA degradation process.

As previously reported, the cytoplasmic assembly of RNA pol II is a sequential process, during which different subassembly complexes associate, in which Rpb4/7 likely bind in the final step, as assumed for the similar process described for bacterial RNA pol [32,37,72]. Our results provide a novel insight into the function of Rtr1, mediating RNA pol II biogenesis, and influencing the assembly of Rpb4 (and probably also the dimer Rpb4/7) with the enzyme, which probably takes place in the cytoplasm, in addition to the previously proposed function for Rtr1 and its human orthologue RPAP2 as nuclear import factors involved in RNA pol II biogenesis [45,51]. These results agree with the model previously reported in eukaryotes, assuming a similar process described for bacterial RNA pol [72], that proposes the cytoplasmic assembly of RNA pol II as a sequential process, during which different subassembly complexes are associated, with the dimer Rpb4/7 being associated in a final step [32].

In agreement with a functional relation between Rtr1 and Rpb4/7, RNA pol II purification using Rtr1-TAP as bait results in the isolation of a 10-subunit with the relative depletion of this dimer [40]. These results do not contradict those presented herein and could also support the notion that Rtr1 is necessary to favor Rpb4 (and likely Rpb7) assembly in the cytoplasm, or even in the nucleus, as Rtr1 interacts with both unmodified and phosphorylated RNA pol II [46,48]. *rtr1Δ* shows genetic interactions with *rpb4Δ* and *rpb7* mutations ([46] and our results), but also with the *rpb1* and *rpb6* mutations that affect integrity or RNA pol II, and lead to the dissociation of either Rpb4 or the dimer Rpb4/7 [62]. Note that Rpb6 and Rpb4/7 interact and that some *rpb6* mutants contain an unstable Rpb4/7 dimer [2,3,62,65,69]. Notably, the growth phenotypes of these double mutants are observed under stress conditions and at high temperatures, which probably reflects the essential role that Rpb4 plays in *S. cerevisiae* under adverse conditions, yet is dispensable under permissive conditions [1,66,73,74,75,76], and that Rpb7 plays an important role in cell survival and stress tolerance [77]. Consequently, these results point to an important function of Rtr1 under stress conditions when Rpb4 and Rpb7 are indispensable.

Accordingly, our results reveal that the lack of Rtr1 affects the assembly of the dimer Rpb4/ Rpb7 to the enzyme and the amount of chromatin-associated RNA pol II containing Rpb4. Since RNA pol II lacking Rpb7 is not functional [70], these results might indicate a preferential nuclear shuttling of functional RNA pol II (both complete and lacking Rpb4), which engage in transcription. In addition, the observed cytoplasmic Rpb4 and Rpb1 accumulation under *RTR1* deletion falls in line with previous observed results for Rpb1 in yeast [45] and human cells under RPAP2 silencing [51], with previous results showing no significant large population of the free heterodimer Rpb4/7 in wild-type cells [40], and with Rpb7 being required for the nuclear import of Rpb4 [78]. Nevertheless, our results do not rule out the nuclear association of Rpb4 with the rest of the enzyme. Furthermore, we can speculate about the possible nuclear diffusion of Rpb4 to associate with nuclear RNA pol II, according to previous propositions [39,45]. To investigate RNA pol II biogenesis some groups have used benomyl, a drug that promotes the depolarization of microtubule and that blocks the nuclear import of RNA pol II, both in yeast and human cells [39,42]. Although this compound could be useful to explore the localization of Rpb4 assembly with the RNA pol II in more detail, our results show that benomyl addition had a clear impact on RNA pol II assembly (Figure S4), thus raising doubts about using this drug to study RNA pols biogenesis.

Our data suggest that the accumulation of chromatin-associated RNA pol II lacking Rpb4 in the absence of Rtr1 seems not to result from a global increase in Rpb4/7 dissociation from the rest of RNA pol II once interacting during transcription [40], although it was proposed that Rtr1 and other elongation factors may influence this process [40]. However, we cannot rule out that this phenomenon may occur for a specific group of genes, in line with a previous proposition [40]. Nevertheless, the occurrence of Rpb4/7 dissociation is controversial [6,10,40].

Our data also suggest that Rtr1 may cooperate with the prefoldin-like Bud27 for the correct association of Rpb5 and Rpb6 with the rest of the enzyme, as the assembly of these two subunits with the RNA pol II is mediated by Bud27 [42]. Notably, the fact that the lack of Bud27 does not mainly affect Rpb4 association with the RNA pol II [42] reinforces the specific role of Rtr1 in Rpb4/7 assembly. In agreement, we observed genetic interactions between *RTR1* and *BUD27* (Figure S5), which are also described in the *Saccharomyces* GENOME DATABASE.

Previous results demonstrated a role for Rpb4 (and also for dimer Rpb4/7) in the life cycle of mRNA by imprinting it cotranscriptionally, and later accompanying it during the export to the cytoplasm, translation, mRNA decay or cytoplasmic accumulation. Furthermore, lack of Rpb4 increases mRNA stability [13,14,15,17,68,71]. Similarly, increased mRNA stability by *RTR1* deletion was observed herein. We propose that increased mRNA stability could be the consequence of reduced Rpb4-mRNA imprinting, due to lower levels of Rpb4 bound to the chromatin-associated RNA pol II. In line with this, mutations that alter the RNA pol II assembly and that alter Rpb4 binding with the rest of the complex also increase mRNA stability by decreasing Rpb4-mRNA imprinting [13,17,62,68]. In agreement, *RPB4/7* overexpression partially suppresses the mRNA stability increase provoked by the *rpb6^Q100R^* mutation that affects the Rpb4/7 association with the rest of the enzyme [17]. Our data also point out that Rpb4-bound to chromatin-associated RNA pol II is a key element in Rpb4-mRNA imprinting, which coincides with previous propositions [14], as lack of Rtr1 does not alter the global Rpb4 amount, but increases the fraction of the free Rpb4 subunit.

In addition, our results point to a specific role for Rtr1 in mRNA decay, which falls in line with previous data reported for this protein autoregulating the decay of its own mRNA [61]. Strikingly, our results suggested that Rtr1 may imprint a broad population of mRNAs, possibly in cooperation with Rpb4. This process may occur likely cotranscriptionally, as lack of Rpb4 reduces the amount of Rtr1 associated with chromatin and decreases Rtr1-mRNA imprinting. We propose that Rpb4 cotranscriptionally modulates Rtr1-mRNA imprinting. Considering the role of Rtr1 as a Ser5-P phosphatase [48,50] and the involvement of Rpb4/7 in the RNA pol II conformational change that occurs not only in yeast, but also in other organisms, favoring elongation [79,80,81], Rtr1-mRNA imprinting may occur during the transition from transcription initiation to elongation. However, we cannot rule out that this imprinting occurs later during transcription elongation as Rtr1 occupies the whole gene body during transcription and coincides not only with RNA pol II phosphorylated at Ser5P, but also at Ser2P [48]. Coinciding with this possibility, Rtr1 physically interacts with different transcription elongation factors, such as Dst1, Spt5, Rba50, and some subunits of the PAF complex [54]. Furthermore, Rtr1-mRNA imprinting may occur during the transition from transcription elongation to termination if we bear in mind the proposed role of Rtr1 in transcription termination [70], and also as CTD Tyr1-P phosphatase [50], and similarly that of human orthologue RPAP2 by participating in pre-mRNA 3′-end formation [57]. As the role of Rtr1 mediating the decay of its own mRNA involves the RNA helicase Dhh1 and exonucleases Rex2 and Rex3 [61], we can speculate that Rtr1 could cooperate with these elements, or others, in global mRNA decay regulation and that this process could include Rpb4.

Our results on mRNA stability come from the addition of thiolutine to inhibit RNA pol II activity, a methodology used to measure mRNA stability in different organisms [82,83,84,85]. In *S. cerevisiae* thiolutine affects RNA pol II in a concentration-dependent manner and high doses of this compound has additional effects on cell physiology [82]. Therefore, we used the concentrations that was described to block RNA pol II activity without significant secondary effects [82]. Notably, recent studies demonstrate the effect of thiolutine on early steps of RNA pol II transcription, leading to the arrest of the enzyme and affecting elongation, probably by impacting the conformation of the RNA pol II clamp (preprint, [86]). As in *S. cerevisiae*, but also in other species, thiolutin affects multiple cellular pathways, such as proteasome activity, glucose metabolism or oxydative stress response, among others [82,87,88,89], we cannot rule out side effects due to thiolutine addition. However, it should not significantly alter mRNA stability in our analyses, as the results obtained corroborated the described increase in mRNA stability caused by the absence of Rpb4, since the lack of Rtr1 affects the association of Rpb4 to the RNA pol II [13,14,15,17,68,71].

**Figure 8:**
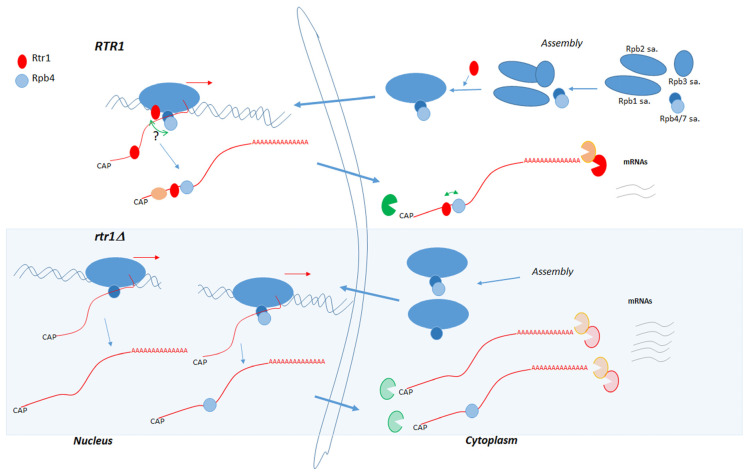
Model for Rtr1 role in RNA pol II cytoplasmic assembly and for Rtr1 and Rpb4 cooperation in mRNA decay. In cytoplasm, Rtr1 would act in RNA pol II assembly to facilitate association Rpb4/7 subassembly complexes in final RNA pol II biogenesis steps. Complete RNA pol II, or that lacking Rpb4 (under *RTR1* deletion), would be transported to nucleus, likely by an Iwr1-dependent pathway. We cannot rule out that Rtr1 also acts by promoting nuclear association of Rpb4 with rest of enzyme. In the nucleus, Rtr1 would associate with RNA pol II and would cotranscriptionally imprint mRNA in cooperation with Rpb4, which also imprints mRNA cotranscriptionally. *RTR1* deletion would affect RNA pol II biogenesis and Rpb4 association with rest of enzyme, as well as transcription mediated by RNA pol II, and Rtr1- and Rpb4-mRNA imprinting. Consequently, lack of Rtr1 would lead to altered imprinted mRNAs, which would be exported to cytoplasm and would increase their mRNA stability by altering actions of 3′ and 5′ mRNA degradation machinery.

As such, we propose that the functional cooperation of Rtr1 and Rpb4 could operate to modulate mRNA decay by connecting transcriptional and mRNA degradation machinery as elements of the crosstalk between mRNA synthesis and decay [18], as we describe in the proposed model in Figure 8. Rtr1 acts in RNA pol II assembly to allow the correct cytoplasmic assembly of Rpb4/7 in a final RNA pol II biogenesis step. The whole RNA pol II would later be imported to the nucleus. Furthermore, we cannot rule out that Rtr1 favors Rpb4 association with the rest of the enzyme in the nucleus. However, the lack of Rtr1 would affect RNA pol II assembly and lead to obtaining both the complete enzyme and an RNA pol II lacking Rpb4, which would be associated with chromatin. During transcription, Rtr1 would associate with RNA pol II (and, thus, with chromatin) in an Rpb4-dependent manner. Rpb4 would cooperate with Rtr1 for cotranscriptional mRNA imprinting, in line with Rpb4 cooperating with the RBP Puf3 to imprint and modulate mRNA stability for a subset of genes [14]. Consequently, Rtr1-Rpb4- mRNA imprinting would allow the correct mRNA decay regulation in the cytoplasm by the action of 3′ and 5′ polyA-mRNA degradation machinery. Under *RTR1* deletion, mRNA imprinting would be altered and affect cytoplasmic mRNA decay, which would consequently increase mRNA stability.

Our data suggest that Rtr1 regulates the decay of a large mRNA population instead of only its own mRNA [61]. Consequently, it would be interesting to define this Rtr1-associated mRNA population. The possibility of this matching at least part of the Rpb4 one, as is the case for Rpb4 and RBP Puf3 [14], would allow us to further analyze the specific role of Rtr1 in mRNA decay regulation in detail. Finally, investigating whether other RNA pol II CTD phosphatases and kinases would also participate in mRNA decay would involve including new layers in the crosstalk between mRNA synthesis and decay.

## 4. Materials and Methods

### 4.1. Yeast Strains, Genetic Manipulations, Media, and Genetic Analysis

The common yeast media, growth conditions and genetic techniques were used as described elsewhere [90]. Formamide sensitivity was tested using a 2% dilution as previously described [46]. Yeast strains and plasmids are listed in Supplementary Tables S1 and S2, respectively [91,92,93,94,95].

The strains containing an *rtr1Δ::KanMX4* allele in our working yeast backgrounds were obtained by chromosomal integration of a PCR product amplified using genomic DNA from strain YFN161 containing the *rtr1Δ::KanMX4* construction as templates (haploid strain derived from the Y26137 diploid strain, EUROSCARF) with oligonucleotides Rtr1-501 and Rtr1-301 (Table S3). The YFN556 strain containing an *rtr1Δ::kanMX4::HIS3* allele was obtained by integrating the *HIS3* maker from plasmid M4754 into the *rtr1Δ::KanMX4* marker of the YFN160 strain by chromosomal integration. Other strains containing the *rtr1Δ::kanMX4::HIS3* allele in our working yeast backgrounds, except YFN562, were obtained by the chromosomal integration of a PCR product amplified using genomic DNA from strain YFN556 with oligonucleotides Rtr1-501 and Rtr1-301 (Table S3). *rtr1::TAP::HIS3MX6* in our working yeast backgrounds resulted from the chromosomal integration of a PCR product amplified using genomic DNA from a strain containing *rtr1::TAP::HIS3MX6* (Open Byosystem) with oligonucleotides Rtr1-501 and Rtr1-301, respectively, as templates (Table S3).

### 4.2. Protein Extract Preparation and Immunoprecipitation

Whole-cell protein extracts and protein immunoprecipitation were performed as described [62]. Briefly for protein extract preparation, 150 mL of cells growing exponentially (OD_600_ 0.6–0.8) were centrifuged, pelleted, and resuspended in 0.3 mL of lysis buffer (50 mM HEPES [pH 7.5], 120 mM NaCl, 1 mM EDTA, 0.3% 3-[(3-cholamidopropyl)-dimethylammonio]-1-propanesulfonate (CHAPS)) supplemented with 1X protease inhibitor cocktail (Complete; Roche; Basel, Switzerland), 0.5 mM phenylmethylsulfonyl fluoride (PMSF), 2 mM sodium orthovanadate, and 1 mM sodium fluoride. Cell disruption was carried out by vortexing (3 cycles, 5 min each) at 4 °C using 0.2 mL of glass beads (425–600 µm; Sigma, Darmstadt, Germany). For the Rpb3 immunopurification, 1 µg of anti-Rpb3 antibody (anti-POLR2C;1Y26, Abcam, Cambridge, UK) was coupled to 40 µL of Dynabeads Sheep-anti-Mouse IgG (Invitrogen, Waltham, MA, USA) per sample, and 2 mg of a whole-cell protein extract were used for each immunoprecipitation. The affinity-purified proteins were released from the beads by boiling for 10 min and were analyzed by Western blotting with different antibodies.

### 4.3. SDS-PAGE, Western Blot Analysis and Immunoreactive Bands Quantification

Protein electrophoresis and Western blot were carried out as described in [62].

For the Western blot analyzes, anti-CTD [63], anti-Rpb3 (anti-POLR2C;1Y26, Abcam), anti-Rpb4 (Pol II RPB4 (2Y14); Biolegend, San Diego, CA, USA), anti-Rpb5 (a polyclonal antibody generated against *S. cerevisiae* Rpb5 in our lab), anti-Rpb6 (a gift from M. Werner), anti-Rpb7 (Rpb7 (yN-19); Santa Cruz Biotechnology, Dallas, TX, USA), antiphosphoglycerate kinase, Pgk1 (22C5D8; Invitrogen), anti-H3 (ab1791; Abcam), antihemaglutinin (anti-HA; 12CA5; Roche) and PAP (Sigma) antibodies were used.

Intensities of immunoreactive bands on Western blots were quantified by densitometry using the software IMAGE STUDIO LITE from images acquired with an office scanner.

### 4.4. Fluorescence Microscopy

The cells expressing Rpb1-Gfp and Gfp-Rpb4 were grown at 30 °C in SD medium supplemented with the corresponding amino acids to reach an OD_600_~0.5–0.7. For Rpb4 localization, strains were transformed with the centromeric vector expressing a Gfp-Rpb4 fusion protein [15] (Supplementary Table S2). Rpb8-ECFP expressing cells were gown in YPD medium with or without being treated with 60 µg/mL of benomyl for 3 h at 30 °C. Slides were covered with Vectashield mounting solution (Vector Laboratories, San Francisco, CA, USA). Fluorescence intensity was scored with a fluorescence microscope (Olympus BX51, Tokyo, Japan).

### 4.5. Preparation of Yeast Cytoplasm and Chromatin-Enriched Fractions

Preparation of chromatin-enriched fractions was carried out by the yChEFs procedure [63,64] using 75 mL of the YPD cultures grown exponentially (OD_600_~0.6–0.8). The chromatin-bound proteins were resuspended in 1X SDS-PAGE sample buffer, boiled for 10 min and analyzed by Western blotting with different antibodies.

Preparation of cytoplasm-enriched fractions for immunoprecipitation experiments was carried out by the yChEFs procedure [63,64] using 150 mL of the YPD cultures grown exponentially (OD_600_~0.6–0.8). The cytoplasm-enriched fraction corresponds to the S2 fraction [63]. The P3 fraction was used as the chromatin-enriched fraction [63].

### 4.6. Isolation of the mRNA-Associated Proteins

mRNA crosslinking was carried out as described elsewhere [13] with some modifications. Briefly, 250 mL of cell cultures grown in SD medium (with requirements) to an OD_600_~0.6–0.8 were exposed to 1200 mJ/cm^2^ of 254 nm UV in a UV crosslinker (Biolink Shortwave 254 nm), resuspended in 350 µL of lysis buffer (20 mM Tris pH 7.5, 0.5 M NaCl, 1 mM EDTA, 1X protease inhibitor cocktail [Complete; Roche]) and 200 µL of glass beads (425–600 µm, Sigma) and broken by vortexing for 15 min at 4 °C. An aliquot of lysate was used as the INPUT control. Lysate was incubated with 150 µL of oligo (dT)_25_ cellulose beads (New England BioLabs, cat no. S1408S) for 15 min at room temperature. PolyA-containing RNA isolation and elution were carried out as previously described [13]. The mRNA-associated proteins were analyzed by SDS-PAGE and Western blot with the appropriate antibodies.

### 4.7. mRNA Stability Analysis

mRNA stability analysis was performed as previously [13]. Briefly, 150 mL of cells were grown in SD (with requirements) to reach an OD_600_~0.5. Cells were treated with 5 μg/mL of thiolutin. Next 15-mL aliquots of cell samples were taken at different times after thiolutin addition (up to 100 min), then pelleted and frozen. Total RNA was isolated from these samples and cDNA was synthesized as previously described [62]. The mRNA stability (half-lives) for the selected genes was analyzed following the decay curves obtained by RT-qPCR with specific primers for the corresponding genes (see Table S3).

For total mRNA half-live calculation, the previously obtained RNA was used for dot-blot analysis: 2 µg of total RNA dots were added to a nitrocellulose membrane previously washed with SSC 2X buffer (SSC 20X: NaCl 3M, sodium citrate 300 mM, pH 7). The membrane was incubated for 5 min at 65 °C and exposed to 400 mJ/cm^2^ of 254 nm UV in a UV crosslinker (Biolink Shortwave 254 nm). After crosslinking, the membrane was incubated in prewarmed hybridisation buffer (NaPO4 0.5 M, pH 7, EDTA 10 mM, SDS 7%) at 56 °C for 45–60 min. Then, 50 pmol of oligodTCy3 were added and incubated overnight at the same temperature. The membrane was washed six times at room temperature using prewarmed washing buffer (NaPO4 0.28 M, pH 7.2, SDS 7%). Fluorescence was analyzed with a Molecular Imager VersaDoc™ MP system at 635 nm and the Quantity one software. The graphical quantifications of the dot blot are shown and represented on a natural logarithmic scale.

The analysis of *GAL1* and *GAL10* genes mRNA stability was performed as previously described [13] by shifting the cells that grew exponentially (OD_600_~0.5–0.6) from SD-galactose to SD-glucose to stop transcription. Cell samples were collected at different time points after glucose addition (5, 10, 15, 20, and 30 min). RNA extraction was performed as described above. mRNA stability was analyzed by RT-qPCR with specific primers for the corresponding genes (see Table S3).

### 4.8. Quantitative Real-Time PCR (RT-qPCR)

Real-time PCR was performed in a CFX-384 Real-Time PCR instrument (BioRad, Hercules, CA, USA) with the EvaGreen detection system ‘‘SsoFast™ EvaGreen^®^ Supermix’’ (BioRad). Reactions were performed using cDNA corresponding to 0.1 ng of total RNA in 5 µL of total volume. Each PCR reaction was performed at least three times with three independent biological replicates to obtain a representative average. The 18S rRNA gene was used as a normalizer. The employed oligonucleotides are listed in Supplementary Table S3.

### 4.9. Chromatin Immunoprecipitation

The chromatin immunoprecipitation experiments were performed using anti-Rpb3 (anti-POLR2C;1Y26, Abcam) or anti-Rpb4 (Pol II RPB4 (2Y14); Biolegend, San Diego, CA, USA) as previously described [62]. For real-time PCR, a 1:100 dilution was employed for the input DNA and a 1:4 dilution for the immunoprecipitated samples DNA. Genes were analyzed by quantitative real-time PCR in a CFX-384 Real-Time PCR instrument (BioRad) in triplicate with at least three independent biological replicates using SYBR premix EX Taq (Takara). Quantification was performed as indicated in the Figure Legends. The oligonucleotides utilized for the different PCRs are listed in Supplemental Table S3.

## Data Availability

Not applicable.

## Supplementary Materials

The following supporting information can be downloaded at: https://www.mdpi.com/article/10.3390/ijms23042002/s1.
