# Supplementary material for: The Association of Rpb4 with RNA Polymerase II Depends on CTD Ser5P Phosphatase Rtr1 and Influences mRNA Decay in Saccharomyces cerevisiae
Source: Int J Mol Sci. 2022 Feb 11;23(4):2002. doi: 10.3390/ijms23042002 (PMC8875030; doi:10.3390/ijms23042002)
Supplement: Supplementary file 1 [file ijms-23-02002-s001.zip › ijms-1463278-supplementary.pdf]

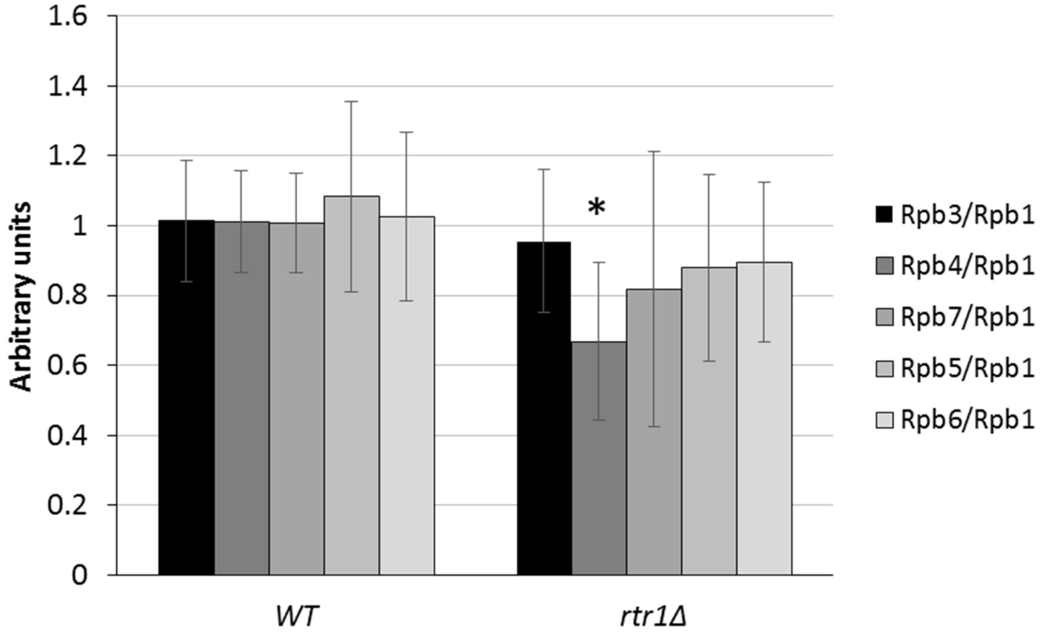

Figure S1

Figure S1: *RTR1* deletion affects the association of Rpb4 to the RNA pol II engaged in transcription. The quantification of the western blot for different RNA pol II subunits *vs.* Rpb1 from chromatin purification, corresponding to the results in Figure 1 for the *rtr1Δ* mutant and wild-type strains. Data are the median and standard deviation of at least three independent biological replicates. *p<0.05 (t-test).

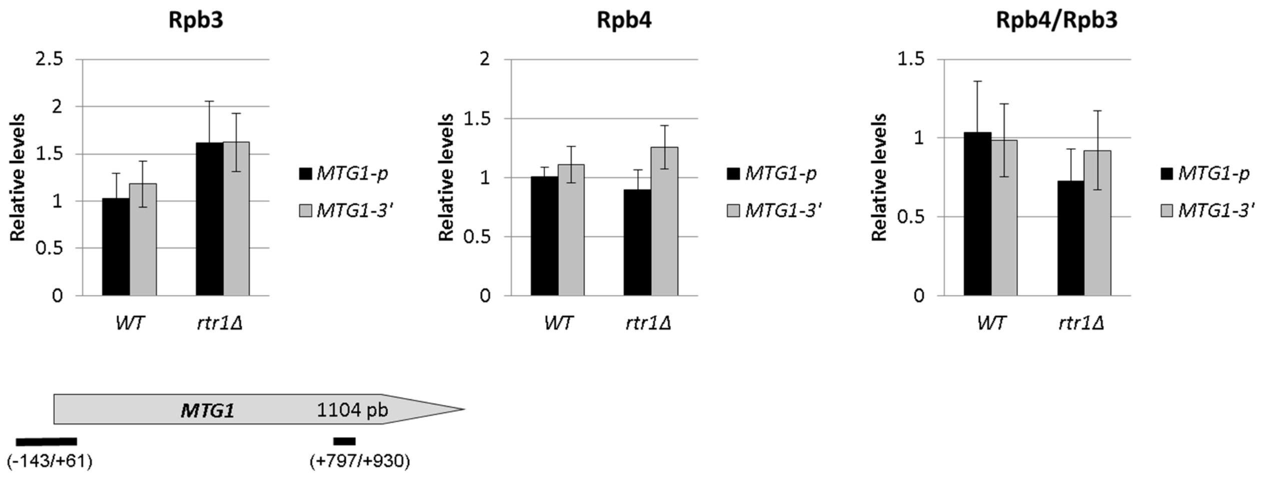

Figure S2: The *rtr1Δ* mutation affects gene occupancy by RNA pol II, but not global Rpb4 dissociation. Chromatin immunoprecipitation (ChIP) analysis for the *MTG1* gene in the wild-type and *rtr1Δ* cells performed with anti-Rpb3 (left panel) and anti-Rpb4 (middle panel) antibodies, against Rpb3 and Rpb4 RNA pol II subunits. Right panel: the Rpb4/Rpb3 ratios for the Rpb4 dissociation analysis, from the left and middle panel's results. Lower panel: the transcription unit used in this work indicating the location of the analysed PCR amplicons. The values found for the immunoprecipitated PCR products were compared to those of the total input, and the ratio of each PCR product of the transcribed genes to a non-transcribed region of chromosome V was calculated.

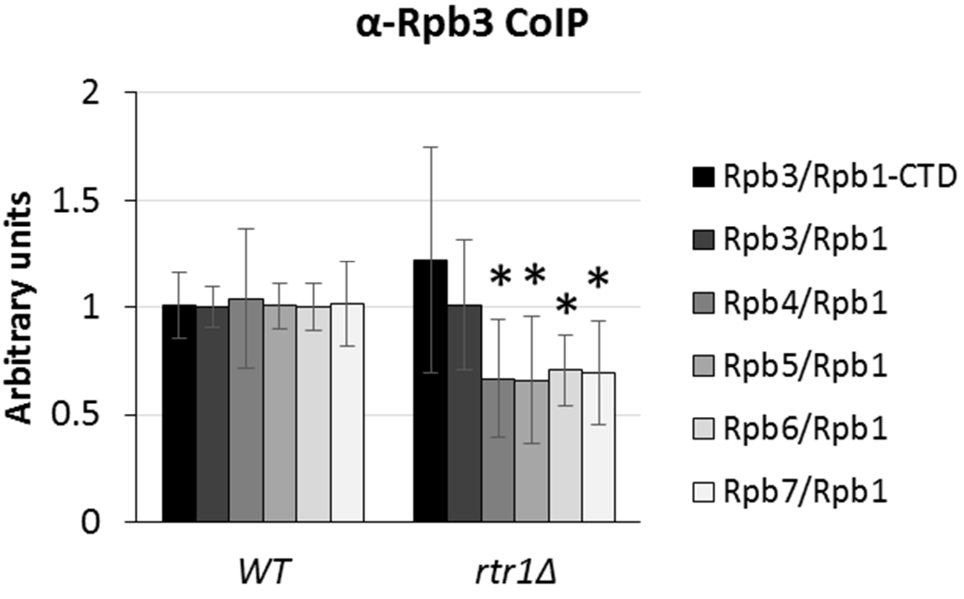

Figure S3

Figure S3: ***RTR1*** **deletion affects the assembly of RNA pol II.**Quantification of the western blot from the RNA pol II immunoprecipitated from the *rtr1Δ* mutant and wild-type strains, from Figure 4A, showing the ratio for each subunit *vs.* Rpb1 (y-80). Data are the median and standard deviation of at least three independent biological replicates. *$p<0.05$ (t-test).

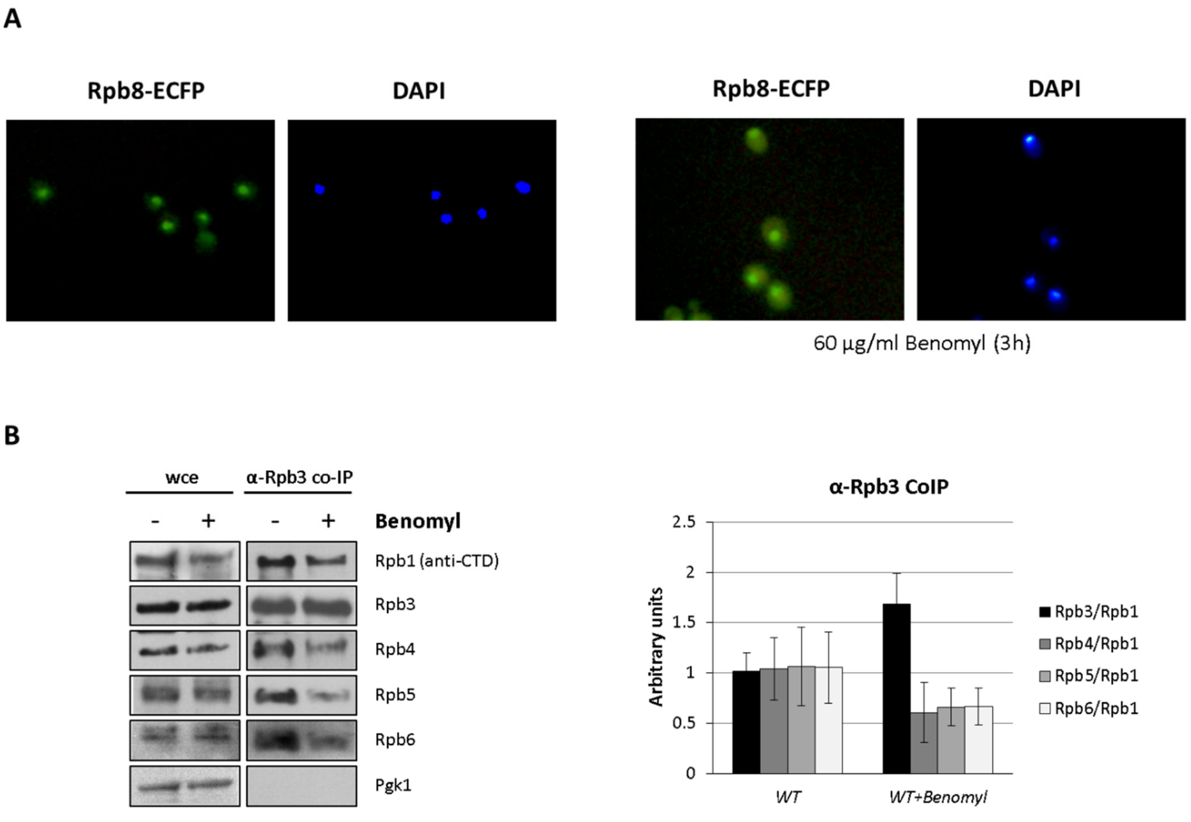

Figure S4

Figure S4: Analysis of RNA pol II cytoplasmic accumulation. A) Rpb8-ECFP (C-terminal ECFP tagged Rpb8) *in vivo* analysis in wild-type cells grown at 30 ºC in YPD and treated with benomyl (60 µg/ml; 3h) shows cytoplasmic Rpb8 accumulation, indicating that nuclear import of RNA pol II is impaired. **B**) RNA pol II immunoprecipitated from a wild-type strain using an anti-Rpb3 antibody, grown in YPD medium at 30ºC with and without benomyl treatment (60 µg/ml; 3h). The left panel shows the western blot analysis of subunits Rpb1 (anti-CTD), Rpb3 Rpb4, Rpb5 and Rpb6 of RNA pol II in whole cell extracts, and in the immunoprecipitated samples. Right panel: quantification of western blot for each subunit *vs.* Rpb1. Pgk1 was tested as a negative control in the RNA pol II purified samples. Graphs represent the median and standard deviation of at least three independent biological replicates.

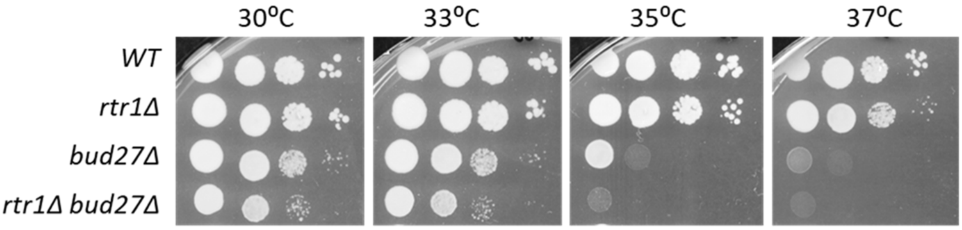

Figure S5

Figure S5: Analysis of the Rtr1 and Bud27 functional relation. Genetic interaction of *rtr1Δ* and *bud27Δ* mutants. Growth of single and double mutants in YPD medium at different temperatures.

Supplementary Table S1. ***Saccharomyces cerevisiae*** **strains**

**Strain**	**Genotype**	**Origin**
BY4741	*MATa his3-Δ1 leu2-Δ0 met15-Δ0 ura3-Δ0*	Euroscarf
Y26137	*MATa/MATα his3-Δ1/his3-Δ1 leu2-Δ0/leu2-Δ0 lys2-Δ0/LYS2 MET15/met15-Δ0 ura3-Δ0/ura3-Δ0 rtr1Δ::kanMX4/RTR1*	Euroscarf
YFN160	*MATα his3-Δ1 leu2-Δ0 ura3-Δ0 rtr1Δ::KanMX4*	This work
YFN161	*MATa his3-Δ1 leu2-Δ0 lys2-Δ0 ura3-Δ0 rtr1Δ::KanMX4*	This work
YFN556	*MATα his3-Δ1 leu2-Δ0 ura3-Δ0 rtr1Δ::kanMX4::HIS3*	This work
Y07202	*MATa his3-Δ1 leu2-Δ0 met15-Δ0 ura3-Δ0 trp1Δ::kanMX4*	Euroscarf
YFN756	*MATa his3-Δ1 leu2-Δ0 met15-Δ0 ura3-Δ0 trp1Δ::kanMX4 rtr1Δ::kanMX4::HIS3*	This work
YFN416	*MATa his3-Δ1 leu2-Δ0 met15-Δ0 ura3-Δ0 rpb1::GFP::HIS5*	[1]
YFN744	*MATa his3-Δ1 leu2-Δ0 met15-Δ0 ura3-Δ0 rpb1::GFP::HIS5 rtr1Δ::KanMX4*	This work
Rpb8-TAP	*MATa his3-Δ1 leu2-Δ0 met15-Δ0 ura3-Δ0 rpb8::TAP::HIS3Mx6*	Open Biosystems
YFN760	*MATa his3-Δ1 leu2-Δ0 met15-Δ0 ura3-Δ0 rpb8::TAP::HIS3Mx6 rtr1Δ::KanMX4*	This work
YFN335	*MATa his3-Δ1 leu2-Δ0 met15-Δ0 ura3-Δ0 lys2-Δ0 rpb8:: ECFP::SpHIS5*	[1]
YFN818	*MATa his3-Δ1 leu2-Δ0 met15-Δ0 ura3-Δ0 lys2-Δ0 rpb8:: ECFP::SpHIS5 rtr1Δ::KanMX4*	This work
YFN106	*MATα his3-Δ1 leu2-3,112 lys2-801 met15-Δ0 trp1-Δ63 ura3-Δ0 bud27Δ::KanMX4*	[1]
Rtr1-TAP	*MATa his3-Δ1 leu2-Δ0 met15-Δ0 ura3-Δ0 rtr1::TAP::HIS3Mx6*	Open Biosystems
Y01285	*MATa his3-Δ1 leu2-Δ0 met15-Δ0 ura3-Δ0 rpb4Δ::KanMX4*	Euroscarf

PAY749	*MATa his3-Δ1 leu2-Δ0 met15-Δ0 ura3-Δ0 dhh1::HA::HIS3*	Gift from Paula Alepuz
YFN725	*MATa his3-Δ1 leu2-Δ0 met15-Δ0 ura3-Δ0 dhh1::HA::HIS3 rtr1::TAP::HIS3MX6*	This work
YFN756	*MATa his3-Δ1 leu2-Δ0 met15-Δ0 ura3-Δ0 dhh1::HA::HIS3 rtr1::TAP::HIS3MX6 rpb4Δ::KanMX4*	This work
YFN562	*MATa his3-Δ1 leu2-Δ0 ura3-Δ0 bud27Δ::KanMX4 rtr1Δ::kanMX4::HIS3*	This work
W303	*MATa ade2-1 can1-100 his3-11,15 leu2-3,112 trp1-1 ura3-1*	[2]
OCSC2115	*MATα ade2-1 can1-100 his3-11,15 leu2-3,112 trp1-1 ura3-1 rpb4Δ::HIS3*	Gift from Olga Calvo
YFN567	*MATa ade2-1 can1-100 his3-11,15 leu2-3,112 trp1-1 ura3-1 rtr1Δ::KanMX4*	This work
YFN638	*MATa ade2-1 can1-100 his3-11,15 leu2-3,112 trp1-1 ura3-1 rpb4Δ::HIS3 rtr1Δ::KanMX4*	This work
WY204	*MATα ade2 his3-Δ200 leu2-3,112 lysΔ201 ura3-52 rpb6Δ::HIS3 + pRP674 (CEN LEU2 rpb6Q100R)*	[3]
Y25602	*MATa/MATα his3-Δ1/his3-Δ1 leu2-Δ0/leu2-Δ0 lys2-Δ0/LYS2 MET15/met15-Δ0 ura3-Δ0/ura3-Δ0 rpb6Δ::kanMX4/RPB6*	Euroscarf
YFN183	*MATα his3-Δ1 leu2-Δ0 lys2-Δ0 met15-Δ0 ura3-Δ0 rpb6Δ::KanMX4 + pFL44L-RPB6 (2µm URA RPB6)*	This work
YFN637	*MATα his3-Δ1 leu2-Δ0 lys2-Δ0 met15-Δ0 ura3-Δ0 rpb6Δ::KanMX4 + pRP674 (LEU2 CEN rpb6Q100R)*	This work
YFN629	*MATα his3-Δ1 leu2-Δ0 lys2-Δ0 met15-Δ0 ura3-Δ0 rtr1Δ::kanMX4::HIS3 rpb6Δ::KanMX4 + pFL44L-RPB6 (2µm URA RPB6)*	This work
YFN634	*MATα his3-Δ1 leu2-Δ0 lys2-Δ0 met15-Δ0 ura3-Δ0 rtr1Δ::kanMX4::HIS3 rpb6Δ::KanMX4 + pRP674 (CEN LEU2 rpb6Q100R)*	This work

YFN517	*MATα ade2-1 his3-Δ200 leu2 lys2 trp1-Δ63 ura3-52 rpb7Δ::LEU2 pGEN-RPB7 (2μm TRP1 RPB7)*	[4]
yBF32	*MATα ade2-1 his3-Δ200 leu2 lys2 trp1-Δ63 ura3-52 rpb7Δ::LEU2 + pGEN-rpb7-ΔC3 (2μm TRP1 rpb7-ΔC3)*	[4]
YFN632	*MATα ade2-1 his3-Δ200 leu2 lys2 trp1-Δ63 ura3-52 rpb7Δ::LEU2 pGEN-RPB7 (2μm TRP1 RPB7) rtr1Δ::KanMX4*	This work
YFN633	*MATα ade2-1 his3-Δ200 leu2 lys2 trp1-Δ63 ura3-52 rpb7Δ::LEU2 + pGEN-rpb7-ΔC3 (2μm TRP1 rpb7-ΔC3) rtr1Δ::KanMX4*	This work

Supplementary Table S2. Plasmids used

Name	Yeast markers and ORI	Origin
M4754	*KanMX::HIS3* disruptor converter	[5]
pCM189	ORI (CEN) *URA3*	[6]
pCM189-*RPB4*	ORI (CEN) *URA3*	[7]
pCM189-*RPB6*	ORI (CEN) *URA3*	This work
pGEN	ORI (2μm) *TRP1*	[8]
pGEN-*RPB7*	ORI (2μm) *TRP1*	[9]
pRS313-GFP-*RPB4*	ORI (CEN) *HIS3*	[10]

Supplementary Table S3. Oligonucleotides used

Gene (or DNA region)	Primer	Sequence
Intergenic region (chromosome V)	IntergChrV-F	TGTTCCTTTAAGAGGTGATGGTGAT
	IntergChrV-R	GTGCGCAGTACTTGTGAAAACC
18S rDNA	18S-501	CATGGCCGTTCTTAGTTGGT
	18S-301	ATTGCCTCAAACTTCCATCG
ACT1	*ACT1*-501	GCCTTCTACGTTTCCATCCA
	ACT1-301	GGCCAAATCGATTCTCAAAA
GAL1	*GAL1*-501	TGGTGTTAACAATGGCGGTA
	GAL1-301	GGGCGGTTTCAAACTTGTTA
GAL10	*GAL10*-501	ACGGAGATTATGGTGCGTTC
	GAL10-301	GGATTTTTGGGGCCTAAGAC
HHT1	*HHT1*-501	TGCCTTTCCAAAGATTGGTC
	HHT1-301	TTGGATAGTGACACGCTTGG
MTG1 (-143/+61 pb)	*MTG1*-503	TCAAAGAACACGGACCATCA
	MTG1-303	TTGGTGTGAAGGATGAAACG
MTG1 (+797/+930 pb)	*MTG1*-502	TGCAAAATTTGAACGATGGA
	MTG1-301	CCACTCGATAGCCGTTGATT
PMA1 (-330/-234 pb)	*PMA1p*-503	AAAGGCCAAATATTGTATTATTTTCAA
	PMA1p-302	TTGGTGTTATAGGAAAGAAAGAGAAA
PMA1 (+9/+116 pb)	*PMA1-5'*-501	ACATCATCCTCTTCATCATCCTC
	PMA1-5'-301	TCAGAAGATTCAGATGCAGCG
PMA1 (+1367/+1532 pb)	*PMA1*-504	GAAGGTTACTGCCGTTGTCG
	PMA1-304	CGGAACCCTCTAGAAGCCAA
PMA1 (+2551/+2757 pb)	*PMA1*-6 (forw)	ATATTGTTACTGTCGTCCGTGTCTGGAT
	PMA1-6 (rev)	ATTAGGTTTCCTTTTCGTGTTGAGTAGA

PYK1 (-37/+52 pb)	*PYK1p*-502	ACAAGACACCAATCAAAACAAA
	PYK1p-301	AGTCAGAACCAGCAACAACG
PYK1 (+254/+352 pb)	*PYK1*-504	CCAAGGGTCCAGAAATCAGA
	PYK1-304	CGTACTTGTCATCGGTGGTG
PYK1 (+769/+950 pb)	*PYK1*-503	CTGACGGTGTTATGGTTGCC
	PYK1-303	TCGGAAACTTCAGCTCTGGT
PYK1 (+1039/+1288pb)	*PYK1*-4 (forw)	CTATGGCTGAAACCGCTGTCATTG
	PYK1-4 (rev)	CAGCTCTTGGGCATCTGGTAAC
RPB4	*RPB4*-502	TGCTTGCAATGGTTCAGAAG
	RPB4-301	CCATTTTTGGTCGAATTTTG
RTR1	*RTR1*-501	TCCGATATTTTTGTCCGAAATAGG
	RTR1-301	GACTCAAAGTGAATTATAGCAAAG
URA2 (-123/+63 pb)	*URA2p*-501	ATATCGGCATCTGGCTTGAA
	URA2p-301	CAGACGGTCACCCGTAGATT
URA2 (+618/+746 pb)	*URA2*-503	GTACGTTCCTCCAGCAGACA
	URA2-303	ACACCCCTTTTGATAAAACAACG
URA2 (+2988/+3213 pb)	*URA2*-501	CGAATTTGATTGGTGTGCTG
	URA2-301	GGCGATGTTGTTGGAAGTTT
URA2 (+6060/+6448 pb)	*URA2*-502	CGCCGCTAAATATTCTCCTG
	URA2-302	CAATTCCGGAGGAGAAACAA
5'-Cy3 labelled probe	oligodTCy3	TTTTTTTTTTTTTTTTTTTTTTTTTTTTTTTTTTT

The primers used were as follows (all 5’-3’)

1. Mirón-García, M.C.; Garrido-Godino, A.I.; García-Molinero, V.; Hernández-Torres, F.; Rodríguez-Navarro, S.; Navarro, F. The prefoldin Bud27 mediates the assembly of the eukaryotic RNA polymerases in an Rpb5-dependent manner. *PLoS Genet.* **2013**, *9*, e1003297.
2. Thomas, B.J.; Rothstein, R. Elevated recombination rates in transcriptionally active DNA. *Cell* **1989,** *56*, 619–630.
3. Tan, Q.; Prysak, M.H.; Woychik, N.A. Loss of the Rpb4/Rpb7 subcomplex in a mutant form of the Rpb6 subunit shared by RNA polymerases I, II, and III. *Mol. Cell. Biol.* **2003**, 23, 3329–3338.
4. Allepuz-Fuster, P.; Martinez-Fernandez, V.; Garrido-Godino, A.I.; Alonso-Aguado, S.; Hanes, S.D.; Navarro, F.; Calvo, O. Rpb4/7 facilitates RNA polymerase II CTD dephosphorylation. *Nucleic. Acids. Res.* **2014**, *42*, 13674–13688.
5. Voth, W.P.; Jiang, Y.W.; Stillman, D.J. New 'marker swap' plasmids for converting selectable markers on budding yeast gene disruptions and plasmids. *Yeast* **2003**, *20*, 985-93.
6. Gari, E.; Piedrafita, L.; Aldea, M.; Herrero, E. A set of vectors with a tetracycline-regulatable promoter system for modulated gene expression in Saccharomyces cerevisiae. *Yeast* **1997,** 13, (9), 837-48.
7. Garrido-Godino, A.I.; García-López, M.C.; García-Martínez, J.; Pelechano, V.; Medina, D.A.; Pérez-Ortín, J.E.; Navarro, F. Rpb1 foot mutations demonstrate a major role of Rpb4 in mRNA stability during stress situations in yeast. *Biochim. Biophys. Acta* **2016**, *1859*, 731–743.
8. Shpakovski, G.V.; Acker, J.; Wintzerith, M.; Lacroix, J.F.; Thuriaux, P.; Vigneron, M. Four subunits that are shared by the three classes of RNA polymerase are functionally interchangeable between *Homo sapiens* and *Saccharomyces cerevisiae*. *Mol. Cell. Biol.* **1995**, *15*, 4702–4710.
9. Zaros, C.; Thuriaux, P. Rpc25, a conserved RNA polymerase III subunit, is critical for transcription initiation. *Mol. Microbiol.* **2005**, *55*, 104–114.
10. Lotan, R.; Bar-On, V.G.; Harel-Sharvit, L.; Duek, L.; Melamed, D.; Choder, M. The RNA polymerase II subunit Rpb4p mediates decay of a specific class of mRNAs. *Genes Dev.* **2005**, *19*, 3004–3016.
